# Telemedicine Research Trends in 2001-2022 and Research Cooperation Between China and Other Countries Before and After the COVID-19 Pandemic: Bibliometric Analysis

**DOI:** 10.2196/40801

**Published:** 2024-08-30

**Authors:** Hanlin Feng, Karin Kurata, Jianfei Cao, Kageyama Itsuki, Makoto Niwa, Atsushi Aoyama, Kota Kodama

**Affiliations:** 1 Graduate School of Technology Management Ritsumeikan University Ibaraki Japan; 2 Course of Information Systems Engineering National Institute of Technology Tsuruoka College Tsuruoka Japan; 3 MergeSystem Inc Fukuoka Japan; 4 Medical Data Science Lab Hoshi University Tokyo Japan; 5 Graduate School of Technology Ritsumeikan University Ibaraki Japan

**Keywords:** telemedicine, telehealth, coauthorship analysis, network analysis, bibliometric analysis, co-occurrence analysis

## Abstract

**Background:**

Advancements in technology have overcome geographical barriers, making telemedicine, which offers remote emergency services, healthcare, and medication guidance, increasingly popular. COVID-19 restrictions amplified its global importance by bridging distances.

**Objective:**

This study aimed to analyze Chinese and global literature data, present new global telemedicine research trends, and clarify the development potential, collaborations, and deficiencies in China's telemedicine research.

**Methods:**

We conducted bibliometrics and network analyses on relevant documents from the Web of Science database from 2001 to 2022. Data collection was completed on October 30, 2023. Considering COVID-19’s impact, 2020 was used as a baseline, dividing the data into 2 periods: 2001-2019 and 2020-2022. The development potential was determined based on publication trends. An international coauthorship network analysis identified collaboration statuses and potential. Co-occurrence analysis was conducted for China and the world.

**Results:**

We identified 25,333 telemedicine-related research papers published between 2001 and 2022, with a substantial increase during the COVID-19 period (2020-2022), particularly in China (1.93-fold increase), moving its global publication rank from tenth to sixth. The United States, the United Kingdom, and Australia contributed 62.96% of the literature, far ahead of China’s 3.90%. Globally, telemedicine research increased significantly post-2020. Between 2001 and 2019, the United States and Australia were central in coauthor networks; post-2020, the United States remained the largest node. Network hubs included the United States, the United Kingdom, Australia, and Canada. Keyword co-occurrence analysis revealed 5 global clusters from 2001 to 2019 (system technology, health care applications, mobile health, mental health, and electronic health) and 2020 to 2022 (COVID-19, children’s mental health, artificial intelligence, digital health, and rehabilitation of middle-aged and older adults). In China, the research trends aligned with global patterns, with rapid growth post-2020. From 2001 to 2019, China cooperated closely with Indonesia, India, Japan, Taiwan, and South Korea. From 2020 to 2022, cooperation expanded to Japan, Singapore, Malaysia, and South Korea, as well as Saudi Arabia, Egypt, South Africa, Ghana, Lebanon, and other African and Middle Eastern countries. Chinese keyword co-occurrence analysis showed focus areas in system technology, health care applications, mobile health, big data analysis, and electronic health (2001-2019) and COVID-19, artificial intelligence, digital health, and mental health (2020-2022). Although psychology research increased, studies on children’s mental health and middle-aged and older adults’ rehabilitation were limited.

**Conclusions:**

We identified the latest trends in telemedicine research, demonstrating its significant potential in China and providing directions for future development and collaborations in telemedicine research.

## Introduction

Development of personal computers, communication technology, and professional medical technology have led to improvements in medical-related technology. Telemedicine provides health care solutions in difficult-to-access areas. Health care organizations worldwide are becoming increasingly interested in implementing telemedicine technologies to improve care and services [1]. Telemedicine is the delivery of health care and the exchange of health care information across distances [2]. Telemedicine dates back to the mid-to-late 19th century [2]. Furthermore, one of the earliest reports was published in the early 20th century, with electrocardiogram data transmitted over telephone lines. Currently known telemedicine was formed in the 1920s to 1960s and was largely driven by military and space technology sectors [2,3]. Moreover, only a few individuals used off-the-shelf commercial equipment at the time [4]. Examples of early telemedicine technology include the use of televisions to facilitate consultations between specialists in institutions and general practitioners in state psychiatric hospitals [5]. Furthermore, it was also used to provide expert medical guidance to airport medical centers from major teaching hospitals [6]. Continued development of internet-based audio and video communication technologies, coupled with high demand for a convenient and efficient way to receive care, enabled the development of telemedicine applications [7]. With the rapid development of telemedicine equipment and information communication technology, telemedicine has developed rapidly and is used widely around the world as a new mode of medical service [8,9]. In particular, in China, telemedicine has been used as a crucial method by the government to address the inequality of medical resources between urban and rural areas [10]. The development of telemedicine in China began in the 1980s. In 1986, the Guangzhou Ocean Shipping Company conducted a cross-sea consultation for emergency patients on the oceangoing freighter through telegraph, which was the earliest telemedicine activity in China. In 1997, the Jinwei Medical Network in China was officially opened to provide remote, off-site, real-time, and dynamic live television consultations for patients with severe illness. Subsequently, the medical institutions at all levels in China began to explore and develop telemedicine. After years of effort, the development of telemedicine entered its golden age. In 2017, 22 provinces in China established telemedicine platforms covering 13,000 medical institutions, providing teleconsultation, telediagnosis, and remote medical education [10].

Travel was restricted owing to the COVID-19 pandemic starting in 2019 [11]. However, telemedicine is attracting attention worldwide as it can overcome the problems caused by distances. Telemedicine was increasingly used during the COVID-19 pandemic as a tool to reduce the spread of potential diseases and fill gaps in health care services. Telemedicine emerged as a crucial tool during the COVID-19 pandemic, playing a significant role in health care delivery and management. The global health crisis prompted by the pandemic accelerated the adoption of telemedicine, highlighting its importance in ensuring the continuity of medical services and reducing the risk of infection transmission. Studies have shown that telemedicine was particularly valuable in providing care to patients while minimizing physical contact, especially in situations where in-person visits may have posed health risks due to the highly contagious nature of the virus [12]. Telemedicine could serve as a public health tool to reduce hospital burden, provide continuity of care, and support disease surveillance and management. Particularly, China took protective measures and relied on telemedicine as a response to the pandemic [13,14]. Zhang and Ma [15] quantified the differences between online and face-to-face delivery of health services during the COVID-19 pandemic through a discontinuous time series study in Beijing, China. Moreover, they compared the impact of COVID-19 on the primary outcomes of face-to-face (outpatient, emergency visits, and discharge) and online (online inquiry) health care services. The impact of COVID-19 on health care for different diseases was also analyzed. The average monthly outpatient and discharge volumes of 22 public hospitals in Beijing dropped by 36.33% (2020: 1,720,180 cases; 2019: 2,701,790 cases) and 35.75% (2020: 21,600 cases; 2019: 86,770 cases), respectively, compared with those in 2019. In addition, the average monthly online consultation volume increased by 90.06% (2020: 12,050 cases; 2019: 6340 cases) [15]. After the COVID-19 lockdown, patients with nonsevere disease opted for online consultations. Conversely, patients with severe disease chose hospital care.

Simultaneously, studies are increasingly focusing on the further development of telemedicine. Although telemedicine technology has developed rapidly around the world, research on China's specific development in this field, the challenges it faces, and the status and potential of international cooperation is still limited. Therefore, understanding the current trends and hot spots of publication in telemedicine is necessary. Understanding the pandemic’s effect on telemedicine research through Chinese protective measures can play a significant role in promoting the development of telemedicine.

In light of this, understanding telemedicine research trends in China, where demand for telemedicine is high, and research relationships among China and other countries is of particular interest. This study aimed to analyze Chinese and global literature data, present new global trends in telemedicine research, and clarify the development potential of China's telemedicine research, potential collaborations, and deficiencies in its own telemedicine research.

Bibliometric analysis is a research method that quantitatively analyzes data from scientific literature and publications. Results of a bibliometric analysis can guide future research by identifying the quality of research and major research areas [16,17]. It includes coauthorship analysis to identify the relationships between institutions and countries [16,17]. Co-occurrence analysis is another methodology used in bibliometric analysis. It investigates co-occurrence relationships between keywords in academic literature and reveals their relationships and structures [16,17]. Co-occurrence analysis allows the identification of research hot spots and the main research directions through keyword clustering. It also allows researchers to obtain information on topics of interest [17-19]. It helps researchers understand the trends in academic research and topics, areas of research focus, and evolution of research fields. Keywords are extracted through co-occurrence analysis [18].

Academically relevant literature on telemedicine bibliometric analyses to date includes the analysis by Armfield et al [20] of 17,932 articles published between 1970 and 2013 in the PubMed database. Furthermore, they divided the time period into 1970 to 1995 and 2009 to 2013. The study examined the themes during the early adoption of telemedicine and compared them with more recently published reports, which provided an understanding of the maturity, scope, and perceptions of telemedicine. Furthermore, they found that the focus of the field shifted from technical issues to clinical applications and assessment.

Sikandar et al [1] conducted a bibliometric analysis that extracted data from 2011 to 2020 from the Scopus database. This study analyzed 1401 articles to examine primary authors, journals, research institutions and countries, and articles with a high number of citations. However, this study lacked data from 2001 to 2011, which was not conducive to an overview of the development of telemedicine. They found that the United States, the Netherlands, and the United Kingdom had many publications in telemedicine. Furthermore, the 10 most studied keywords were identified: mobile health (mHealth), telemedicine, internet, eHealth literacy, technology, self-management, digital health, primary health care, mental health, and electronic health records. This study enabled researchers to understand that telemedicine was an emerging discipline as well as an understudied field. However, telemedicine experienced rapid development after 2020 due to the COVID-19 pandemic. Therefore, updating our understanding regarding telemedicine is essential.

Yanga et al [7] used the Web of Science database to obtain data on telemedicine over a 20-year period, from 1993 to 2012, which produced 7960 publications. Bibliometric analysis revealed that, although the total volume of telemedicine literature increased significantly over the past 20 years, publication activity in each country and region changed over time. The results revealed that the number of telemedicine publications per year increased from 10 in 1993 to 996 in 2012. There was annual growth from 1993 to 2000; however, the growth rate declined between 2000 and 2008. Despite this, growth in telemedicine publications continued to decrease and has remained steady since 2009 [7]. Although the United States leads the cumulative number of telemedicine publications, Norway ranked the highest when ranking countries by publications per capita. The study also found that the annual increase in the number of publications was inconsistent between 1993 and 2012. Moreover, neuroscience and nursing were 2 significant subresearch fields in telemedicine research.

Waqas et al [21] conducted a bibliometric analysis and extracted data through the Web of Science from 2010 to 2019, with a focus on English publications. They found that academic research in telemedicine increased significantly over the 10-year period. Furthermore, research on telemedicine was primarily conducted by institutions in high-income countries. In addition, telemedicine research shifted from radiology to specific disciplines, such as telemedicine, teledermatology, and telecare. Disciplines that promoted joint research included public environment and occupational health, psychiatry, pediatrics, health policy and services, nursing, rehabilitation, radiology, pharmacology, surgery, respiratory medicine, neuroscience, obstetrics, and geriatrics. However, after the COVID-19 pandemic, telemedicine research fostered telemedicine development, which implies the need for further analysis.

Lan et al [14] discussed the research direction of telemedicine during COVID-19, clarified the diseases for which telemedicine technology was used, and identified the medical services it provided. As of December 16, 2021, 5224 telemedicine research papers on COVID-19 were retrieved from the PubMed database. The United States had the most published articles on telemedicine. Simultaneously, most research was related to the provision of health care and mental health services. Although the study mainly explained the application of telemedicine during COVID-19, it did not consider the difference in the timeline before and after the COVID-19 pandemic. Hence, there is a need to further analyze the changes in telemedicine research motivated by COVID-19. Telemedicine’s popularity and acceptance, as well as the awareness and recognition of it by patients and health care professionals, may have changed before and after the COVID-19 pandemic. Clarifying these changes is important when considering the future direction of telemedicine.

Waqas et al [21] also found that Web of Science indexed 6896 papers from 2010 to 2019, with an overall h-index of 87. Furthermore, each study was cited an average of 10.64 times. In total, 42,381 papers were cited 73,354 times. In addition, both the number of published papers and number of citations showed an increasing trend from 2010 to 2019. Regionally, publication output (in English) was highest in high-income countries: the United States, Australia, the United Kingdom, Canada, and Germany. However, 2 middle-income countries, India and China, also featured in the top 10 for publication output. These results demonstrated the rapid growth in telemedicine, even before the COVID-19 pandemic. We anticipated that the research landscape and implementation of telemedicine infrastructure would likely advance exponentially during and after the COVID-19 pandemic. This was echoed by a recent report from the American Medical Association, which predicted that “$250 billion in care could shift to telehealth in the wake of COVID-19, boosting research and infrastructure” [22].

Through preliminary research in telemedicine, we found that the relevant literature increased or decreased every year [1,21]. However, the overall trend was rising. Simultaneously, the United States made the largest contribution to the literature [21]. Research hot spots ranged broadly, including early technology, teleconsultation, telepathology, teleradiology, teledermatology, chronic disease care, and home care.

This study aimed to analyze Chinese and global literature data, present new global trends in telemedicine research, and clarify the development potential of China's telemedicine research, potential collaborations, and deficiencies in its own telemedicine research. By identifying and understanding its own opportunities and shortcomings, China could further develop effective strategies to promote the innovation and development of telemedicine technologies and services. Identifying potential countries with which to collaborate will help China establish international collaboration in telemedicine, share knowledge and experience, and jointly respond to global health challenges, such as COVID-19.

## Methods

### Data Collection

**Figure 1:**
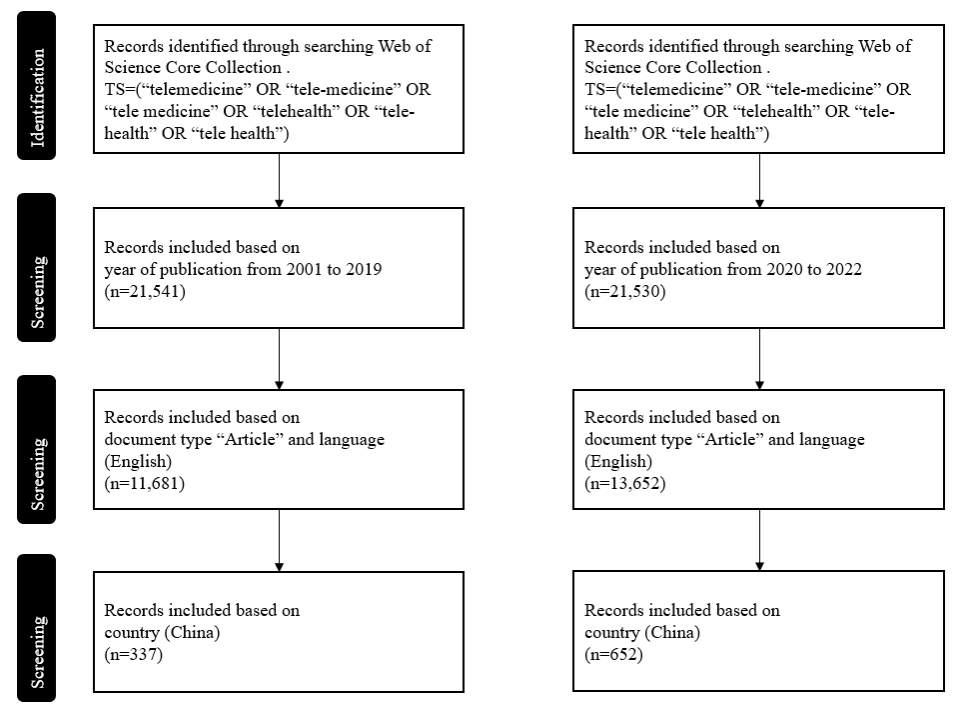
Data collection strategy for the bibliometric analysis of telemedicine research. TS: topic search.

We conducted a comprehensive literature search for telemedicine research articles published between 2001 and 2022 in the Web of Science database. A combination of the following keywords was used, with all relevant keywords in 1 search box: topic search = (“telemedicine” OR “tele-medicine” OR “tele medicine”) or (“telehealth” OR “tele-health” OR “tele health”). This means that we searched for articles containing any “telemedicine”-related terms and any “telehealth”-related terms at once. In this way, we ensured that all results contained relevant keywords in the fields of telemedicine and telehealth. Our selection criteria were limited to articles published in English within a specified time frame. The search was divided into 2 periods: 2001-2019 and 2020-2022. As illustrated in Figure 1, the initial search yielded 11,681 and 13,652 articles in the first and second periods, respectively. Subsequently, we extracted data that pertained to publications from China, which resulted in 337 and 652 articles in the first and second periods, respectively. Data collection was completed on October 30, 2023.

We selected the Web of Science database to extract data for our publication analysis. Since the use and definition of “telemedicine” were not uniformly standardized across the literature and had variations in spelling and terminology, it is critical to encompass such diversity. The term “telemedicine” and its synonymous term “telehealth” were used interchangeably. A previous study highlighted the importance of including both “telemedicine” and “telehealth” in searches for comprehensive representation [7]. This database was chosen for 2 primary reasons. First, it provided extensive citation coverage across more than 20,000 peer-reviewed journals in diverse fields, such as health, social sciences, and engineering. Second, it was the sole database that enabled citation management across 250 distinct subjects [21]. These features led numerous researchers to consider the Web of Science as an optimal resource for conducting bibliometric analyses [23-25].

Before conducting data analysis, it is crucial to ensure the data's accuracy and reliability. To this end, we performed data cleaning to check for and remove possible duplicate entries in the database, ensuring that each article appeared only once. Databases may list multiple institutions as affiliations for a single author due to the author's personal profile, which may not match the affiliation listed in the article. In such cases, we cleaned the entries to retain only the author's valid affiliation at the time of publication. If the affiliation field for the same author contained multiple institutions, we cleaned the data to reflect only the primary affiliation associated with the publication. This step is critical to preventing misrepresentation of institutional contributions and collaborations. Through these data cleaning steps, we ensured the data set's completeness and accuracy, avoiding bias in the research results due to duplicate or erroneous data, including titles, author names, journal names, and publication dates.

### Use of Software

This study used Microsoft Excel (version 2019), VOSviewer (version 1.6.18), R studio (version 3.3.0), Bibliometrix package (version 4.1.3), Statnet package (version 2019.6), and CiteSpace (version 6.2.R4).

### Data Analysis

We used Microsoft Excel (version 2019) to collect the statistics and organize the following literature data [26,27]: the annual number of publications in China and the world, the top 10 countries in the world in total number of publications from 2001 to 2022, the number of publications in these countries from 2001 to 2019 and 2020 to 2022, and magnification. We also used Microsoft Excel (version 2019) to calculate the ratio of the number of publications in each country to the total number of publications and determine the contribution of each country in this field. Magnification is the publication growth ratio in a period, that is, comparing the ratio of the number of publications in the 2 different time periods, 2001-2019 and 2020-2022. The calculation used the following formula: N_2_/N_1_, where N is the number of publications in each time period [28]. This comparison can be used to measure how quickly the telemedicine field or topic is growing or changing over time. This ratio helps understand whether the field of telemedicine has experienced significant research growth during and after the COVID-19 pandemic.

Furthermore, we performed bibliometric analysis with VOSviewer (version 1.6.18). VOSviewer is a software tool designed to construct and visualize bibliometric networks [17]. Bibliometric analysis is the application of mathematics and statistics [16]. We used VOSviewer and generated bibliometric maps to visualize social networks based on geographical data, which thereby showed international collaborations within the telemedicine domain. Additional analyses were performed via R Studio (version 3.3.0) and incorporated the Bibliometrix and Statnet packages to delineate intercountry relationships in telemedicine research. These packages are tools for bibliometric analysis and scientific mapping, respectively. Bibliometrix is an R package specially designed for bibliometric analysis and provides functions for data import, cleaning, analysis, and bibliometric data visualization [29]. Conversely, Statnet is a software package used to describe and visualize networks of relationships between countries in bibliometric data, with a special focus on international research collaborations [30]. These tools are valuable for researchers who conducted bibliometric studies in various fields. They enable the analysis of publication trends, citation networks, coauthorship patterns, and international collaborations and provide insights into the structure and dynamics of scientific research. By leveraging these packages, researchers gain a comprehensive view of scholarly communications and identify key contributors, influential publications, and emerging trends. The use of Bibliometrix and Statnet in R Studio was consistent with the purpose of conducting bibliometric and network analyses to describe international collaborations in telemedicine. These tools facilitated the visualization and interpretation of social networks for academic collaboration, elucidated the relationships between countries, and disseminated research in telemedicine. We established thresholds, setting the minimum numbers of publications in a country and citations to 10 and 5 times, respectively, to ensure the relevance and importance of network analysis. This approach allowed us to identify China’s role and evolution in international telemedicine collaboration [31,32].

**Figure 2:**
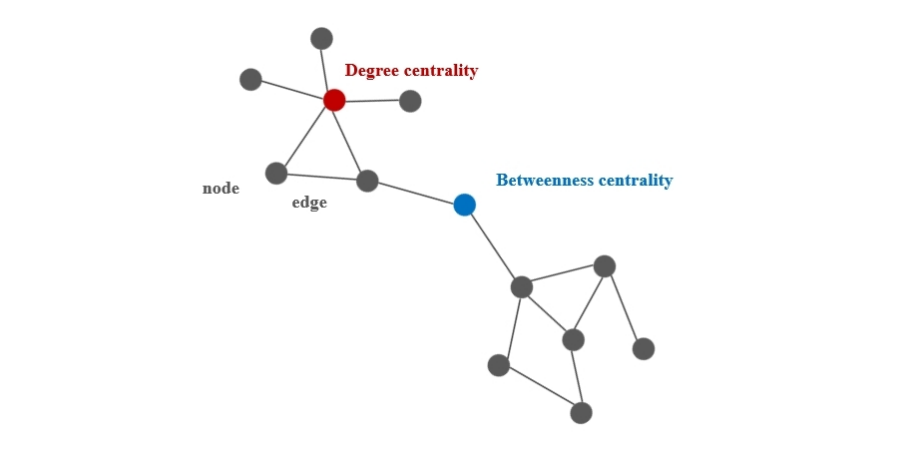
Description of a network diagram.

Figure 2 presents a network diagram in which each point is shown as a node within the network structure. Connections between the points, indicative of relationships, are illustrated as edges. Degree centrality is a key measure within this context and was defined as the number of direct neighbors of a node. It was calculated by adding the total number of direct links that a node had with other nodes [33].

Betweenness centrality quantified a node's role as an intermediary within a network. It was measured by the frequency with which a node occurred on the shortest paths between pairs of nodes. In essence, a node with high betweenness centrality acted as a critical bridge within the network’s shortest path structure. The calculation of betweenness centrality was derived from the aggregate shortest paths that passed through a given node:

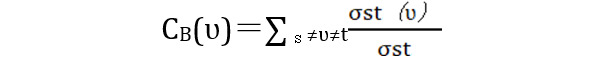



where σst was the total number of shortest paths between nodes s and t and σst(υ) was the number of paths passing through node υ [33,34].

Betweenness centrality is known as an “intermediary agency” role in the network. If one node was in the path to other unique nodes through communicating, connecting, transporting, or trading, then it could be important and is likely to have a high betweenness centrality [35].

We conducted co-occurrence analysis using author-provided keywords in VOSviewer to identify research hot spots in telemedicine. We chose binary counting when using VOSviewer for analysis. This means that each keyword is counted only once, no matter how many times it appears in the document. This is done to ensure that the weight of each keyword in the network graph is not affected by its frequency of occurrence in a single document but is based on its occurrence in multiple documents. The minimum number of co-occurrences was set to 50. This is to ensure that the keywords analyzed are sufficiently representative in international research. For China’s data, since the amount of extracted data was lower, the minimum number of co-occurrences was set to 5. This ensures that important keywords are included even in smaller data sets. Due to the search strategy, the keywords telemedicine and telehealth (and keywords with similar meanings) appear more frequently and occupy a larger weight in the co-occurrence network graph. Such keywords are considered to affect the distribution of the remaining keywords; therefore, when mapping the co-occurrence of keywords in China and the world, the keywords used in the search strategy that appeared in the results are deleted, and the results are concentrated on valuable research topic buzzwords [36]. Keywords were standardized, and synonyms and different spellings were merged. For example, we combined synonyms like “COVID19” and “covid-19” into 1 unified keyword.

To examine the changes in hot spots more comprehensively, we used CiteSpace (Basic version 6.2. R4) to visualize the changes over time for international telemedicine research trends [37]. We used CiteSpace (version 6.2. R4) to analyze keyword bursts. CiteSpace is popular scientometric software to analyze and visualize trends, patterns, and frontiers in scientific literature. It includes several key concepts, such as “keyword burst,” and has 2 important concepts [38]. Keyword explosions refer to sharp increases in the frequency of the use of certain keywords within a specific period. An explosion could indicate that the represented concept, technology, or topic represented had received widespread attention or become a hot research topic during that period. CiteSpace’s (version 6.2.R4) keyword burst analysis helps identify and track research trends and hot areas [38].

## Results

### Telemedicine Research Publications

**Figure 3:**
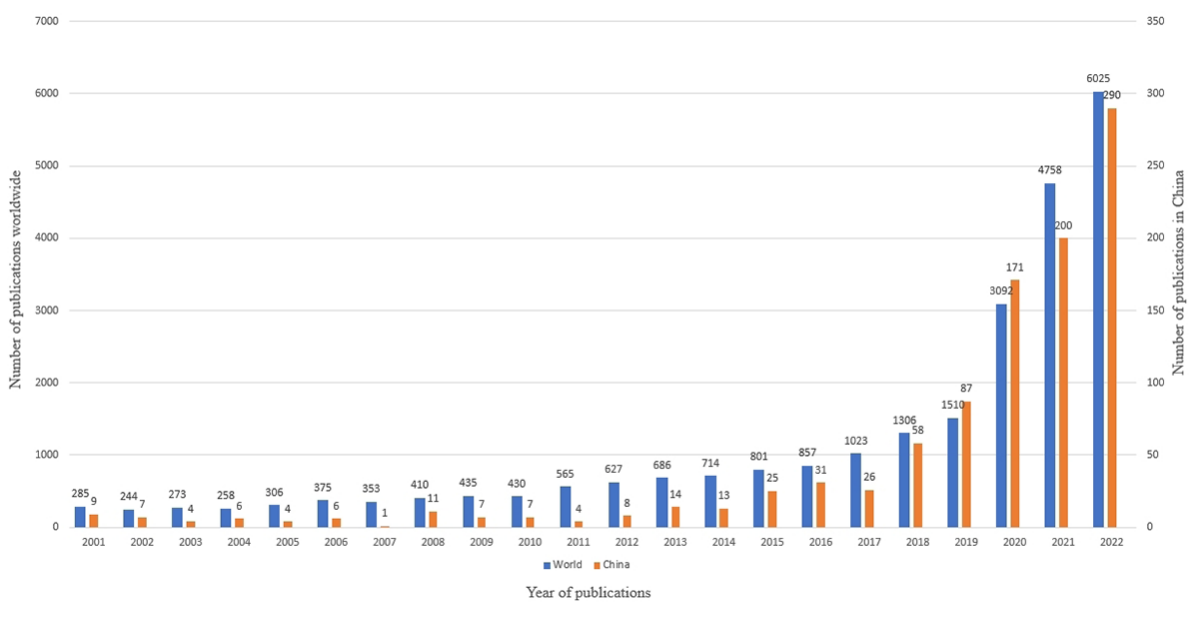
Number of international telemedicine publications.

The publication patterns in telemedicine revealed a global upward trajectory, which was particularly significant over the past 3 years. Although the growth from 2001 to 2019 was gradual, a marked acceleration in publication volume was observed after 2020. The number of telemedicine papers published by the top 10 countries from 2020 to 2022 surpassed those published in the preceding 19 years, as shown in Table 1. This was also notably true for China, where the publication counts from 2020 to 2022 were approximately 1.93 times the aggregate for those in 2001 to 2019. Table 1 also shows the global ranking of Chinese telemedicine research publications. China’s global ranking increased from tenth in 2001 to 2019 to sixth in 2020 to 2022. This surge highlighted the escalating significance of telemedicine, propelled by technological advancements in health care and intensified by the COVID-19 pandemic [20-22]. Figure 3 illustrates the burgeoning global interest in telemedicine research, a trend expected to persist. Figure 3 also charts the trajectory of telemedicine publications in China, which shows a steady increase from 2001 to 2019, followed by a sharp increase in the subsequent years. This pattern mirrored the global trend and highlighted China's intensified focus on telemedicine research in the face of recent health challenges.

**Table 1:** Top 10 countries by publication volume (total number of publications=25,333).

Rank	Country	Publications in the country, n (%)	Publications by time period		Magnification^a^
			2001-2019, n	2020-2022, n	
1	United States	12,112 (47.81)	5295	6817	1.29
2	Australia	2035 (8.03)	1002	1033	1.03
3	England	1804 (7.12)	873	931	1.07
4	Canada	1543 (6.09)	782	761	0.97
5	Italy	1201 (4.74)	485	716	1.48
6	Germany	1022 (4.03)	498	524	1.05
7	India	998 (3.93)	362	636	1.76
8	China	989 (3.9)	337	652	1.93
9	Spain	842 (3.32)	401	441	1.10
10	Netherlands	665 (2.62)	352	313	0.89

^a^Number of publications before and after 2020.

### International Coauthorship Network for the Telemedicine Research Field

**Figure 4:**
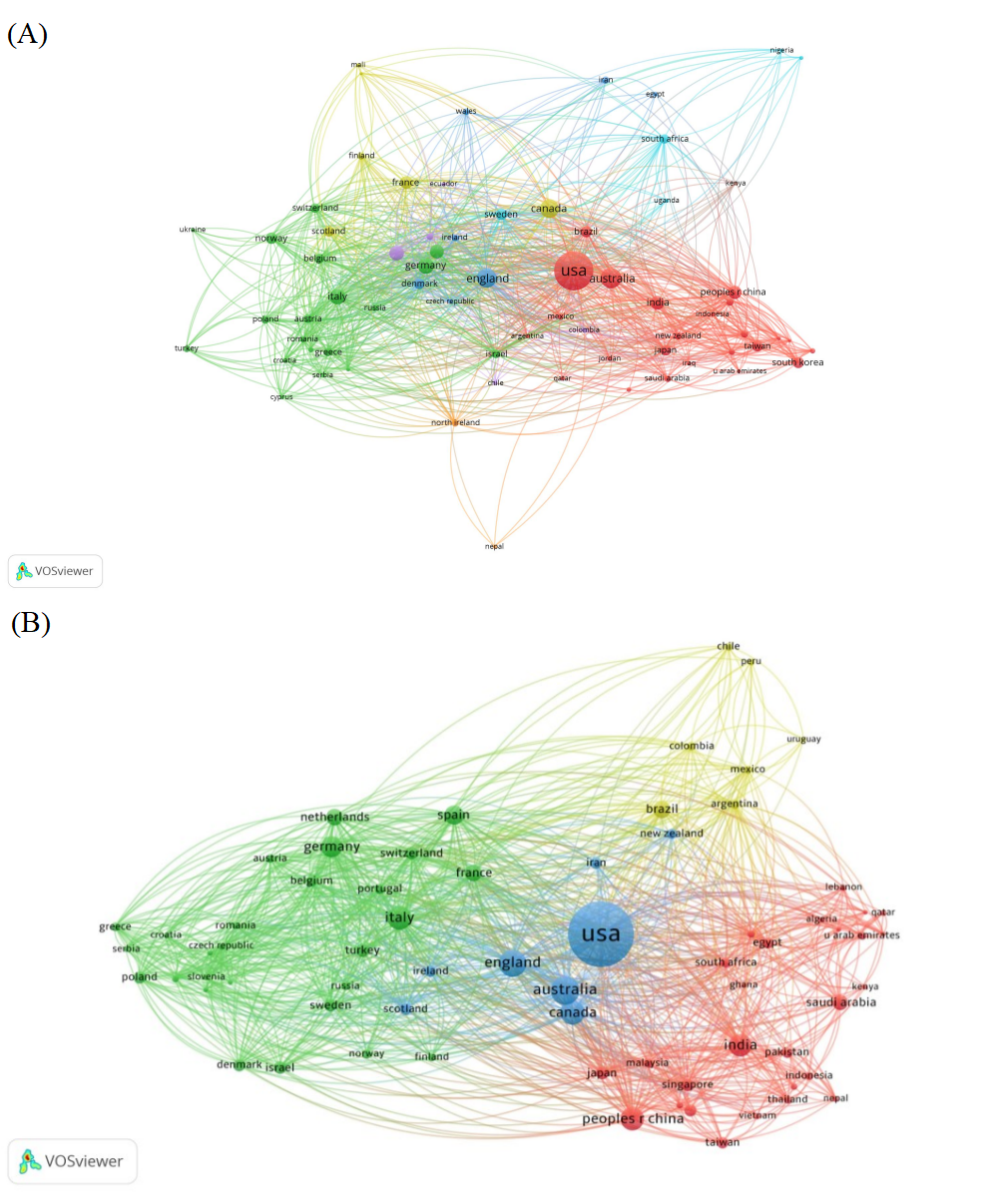
International co-authorship network in telemedicine: (A) 2001-2019 and (B) 2020-2022.

Figures 4A and 4B depict the international coauthorship network in the telemedicine field. The size of each node corresponds to the number of publications in each country, and larger nodes indicate a higher volume. Proximity between 2 nodes suggest the degree of relatedness between the corresponding countries regarding research. An interconnecting line represents collaborative relationships, and thicker lines denote more frequent or substantial collaborative efforts. The different colors assigned by VOSviewer signifies different clusters within the network, where nodes of the same color typically share common properties or characteristics [39]. The network demonstrates the global scale of telemedicine research, showcasing various nations’ contributions and cooperative efforts to improve health care through telemedicine technologies. As shown in Figure 4A, the United States and Australia emerged as central hubs for coauthor networks between 2001 and 2019, which emphasizes their critical roles in telemedicine research collaboration. Although Brazil’s node is relatively small, its proximity to the United States and the network’s core suggests an important collaborative link. China maintained close cooperative relations with Indonesia, India, Japan, Taiwan, and South Korea. China’s main cooperation collaboratives were neighboring Asian countries in the same geographical location. However, network density was more concentrated in Europe and North America, which could reflect more intensive collaboration in these regions.

Figure 4B further illustrates the scale of international collaboration in telemedicine between 2020 and 2022. The United States remained the largest node, which represented the most extensive international collaboration. The countries in the center of the network included the United States, the United Kingdom, Australia, and Canada. Importantly, China maintained close cooperation with countries from Asia including Japan, Singapore, Malaysia, and South Korea. In addition, China, Saudi Arabia, Egypt, South Africa, Ghana, Lebanon, and other African and Middle Eastern countries unified from the different colored clusters in Figure 4A to a common red cluster in Figure 4B. Cooperation with countries in Africa and the Middle East was also strengthening. Simultaneously, European countries unified from the different colored clusters in Figure 4A to a common green cluster in Figure 4B. Italy is in the central area, and the size of the nodes is relatively large, which indicates more contributions to publications.

**Figure 5:**
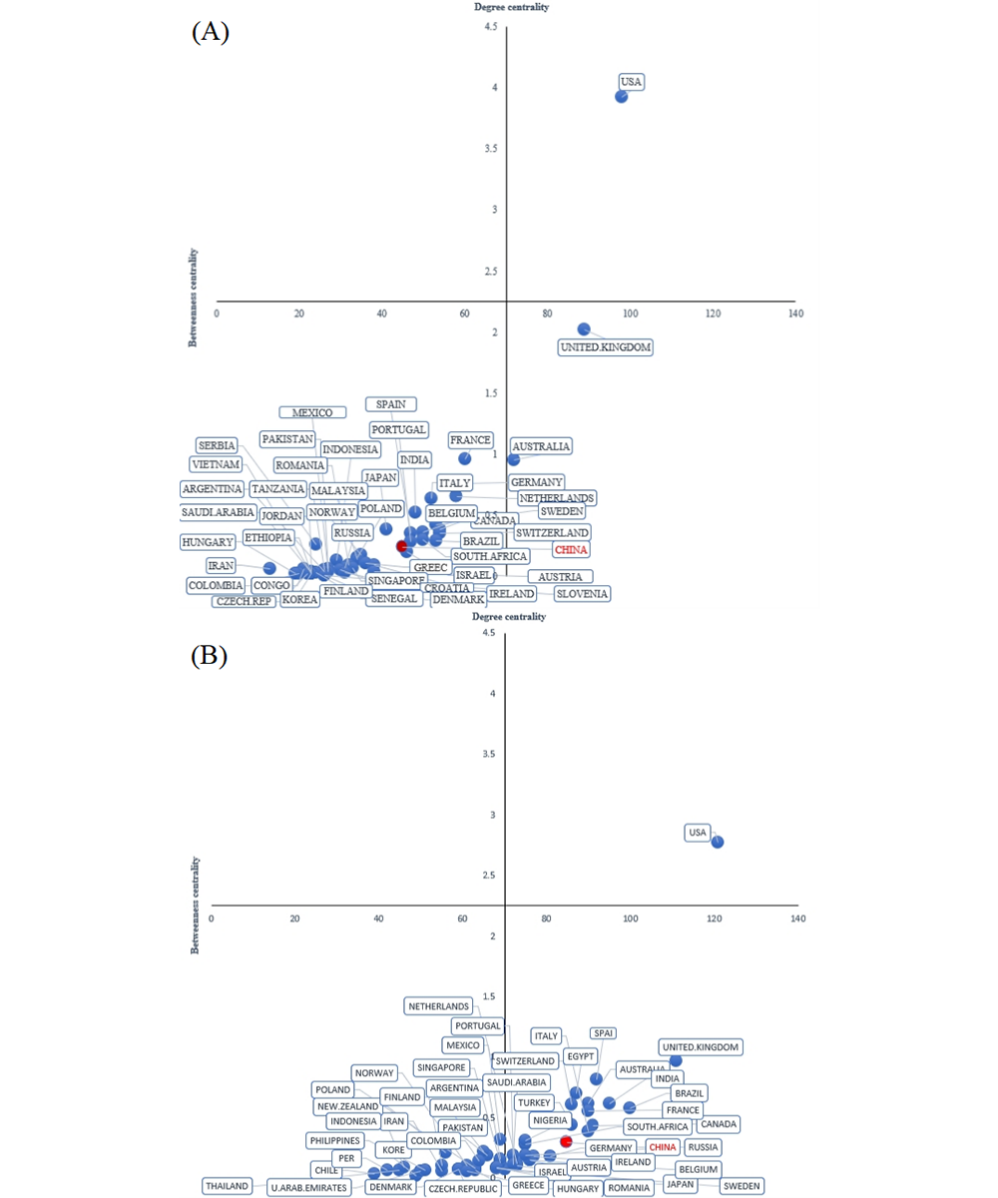
Matrix of degree and betweenness centrality in telemedicine: (A) 2001-2019 and (B) 2020-2022.

This study used RStudio (version 3.3.0) to generate the degree and betweenness centrality for 130 countries in the field of telemedicine. Matrices of the top 50 countries in degree and betweenness centrality from 2001 to 2019 (complete list shown in Multimedia Appendix 1) and 2020 to 2022 (complete list shown in Multimedia Appendix 2) are shown in Figures 5A and 5B, respectively. The central axis divides the matrix into 4 quadrants, with the degree centrality increasing along the X axis from left to right and the betweenness centrality increasing along the Y axis from bottom to top. The X axis quantifies the degree centrality, which reflects the number of connections a country has within the network. In contrast, the Y axis quantifies betweenness centrality, which indicates a country's role as an intermediary within the network.

In Figure 5, the span of the X and Y axes ranged from 0 to 140 and 0 and 4.5, respectively. From 2001 to 2019, shown in Figure 5A, to 2020 to 2022, shown in Figure 5B, the countries as a whole moved to the right and downwards, indicating that countries within the international telemedicine research collaboration network exhibited a general increase in degree and decrease in betweenness centrality. This trend implies that, although the number of international collaborations increased, the role of these countries in the dissemination of research information diminished.

In Figure 5, the United States consistently occupied the quadrant characterized by high degree and betweenness centralities, which indicates its significant role as both a primary collaborator and vital conduit for information flow. In contrast, China was situated in a quadrant of low degree and betweenness centralities from 2001 to 2019. However, from 2020 to 2022, China shifted to the quadrant indicative of high degree centrality but low betweenness centrality, which aligns with the general trend in which international collaborations increased while the centrality in information dissemination decreased [40,41].

### Co-Occurrence Analysis of Author Keywords for China and International Trends in the Telemedicine Research Field

**Figure 6:**
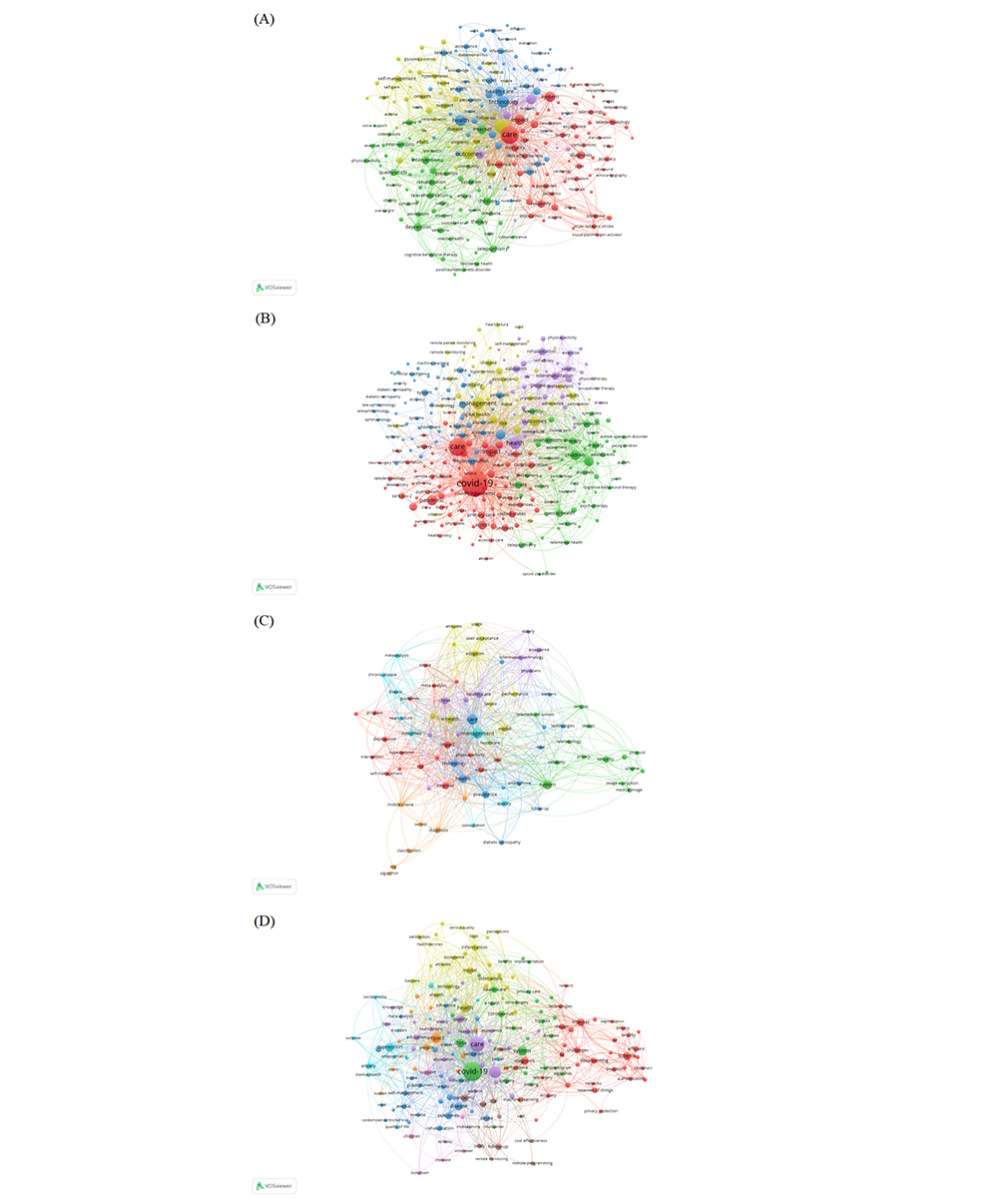
Keyword co-occurrence network in telemedicine in (A) the world in 2001-2019, (B) the world in 2020-2022, (C) China in 2001-2019, and (D) China in 2020-2022.

Figure 6 presents the keyword co-occurrence network. Figures 6A and 6B represent international trends from 2001 to 2019 and 2020 to 2022, respectively. Figures 6C and 6D represent trends in China from 2001 to 2021 and 2020 to 2022, respectively.

In Figures 6A and 6C (2001-2019), the keyword analysis revealed that “care” and “health” were the predominant nodes, which signifies their foundational role in telemedicine research. The prominence of “technology” underscores its critical influence, while the association with terms, such as “mobile health” and “eHealth,” reflects the importance of digital innovation in telemedicine service provision. The emergence of “user acceptance” and “self-management” as notable keywords denotes the sector's focus on consumer engagement and empowerment of patients in managing their health. The keyword “outcomes” highlights the significance of research results and evaluation of service efficacy in telemedicine. Moreover, the keyword “mobile” suggests an intensive research interest in the application of mobile technologies to telemedicine services. “Health information” and “data” highlight the importance of information management and data analysis, and the emergence of “quality of care” underscores a commitment to service excellence.

The focus on “security” and “privacy” in Chinese research indicates a growing awareness of the need for data protection measures in telemedicine services. Comparison of Figures 6A and 6C shows that, although international studies were inclined toward nursing and health, China had a unique focus on fundamental technology and systems management, with a special emphasis on health data collection and analysis. This difference could stem from specific telemedicine policies implemented in China [41]. However, compared with Figure 6A, which shows global keyword co-occurrence, Figure 6C shows that China’s telemedicine research had fewer keywords in mental health.

As seen in Figures 6B and 6D, the keyword landscape from 2020 to 2022 was significantly reshaped by the emergence of the COVID-19 pandemic, with the importance of “COVID-19” and “pandemic.” This was demonstrated in research discourse. These terms underscored the significant impact of health crises on telemedicine research and implementation. The increase in “mental health” keywords may have relationships with the widespread use of telemedicine in mental health services and psychotherapy. In addition, the mental health issues caused by the pandemic may have provided potential opportunities for telemedicine to implement psychological assistance. The prominent appearance of “data,” “analytics,” and “artificial intelligence” demonstrates an accelerated convergence of data analytics and artificial intelligence in telemedicine, which points to technological changes triggered by the pandemic. Additionally, consistent references to “telemedicine,” “technology,” and “digital” reaffirm the centrality of technological innovation in telemedicine services. The emergence of “children” as a keyword highlights the importance of telemedicine for pediatric care. “Patient engagement” and “self-management” suggest that patient autonomy was increasing, which could have been influenced by increased health awareness and limited access to physical care during the pandemic.

Consistent with the global pattern, the term “COVID-19” became the focus of telemedicine research in China during the pandemic. This reflects the significant impact of the pandemic on the health care system and the resulting shift in research focus toward telemedicine. The clustering of terms, such as “eHealth,” “mHealth,” and “digitalization,” highlight the critical role that digital and mobile technologies played in health care delivery in China. The observed increases in the keywords “mental health” and “depression” may indicate an increasing focus in telemedicine research on mental health services and psychotherapy. In addition, the emergence of keywords such as “children” and “rehabilitation” in telemedicine research highlights the growing interest in specialist telemedicine applications. This may reflect an increased awareness of diverse health care needs, including those for the older adult population, pediatric care, and rehabilitation services. These needs may be impacted by the pandemic’s broader impact on health care priorities and service delivery models.

**Figure 7:**
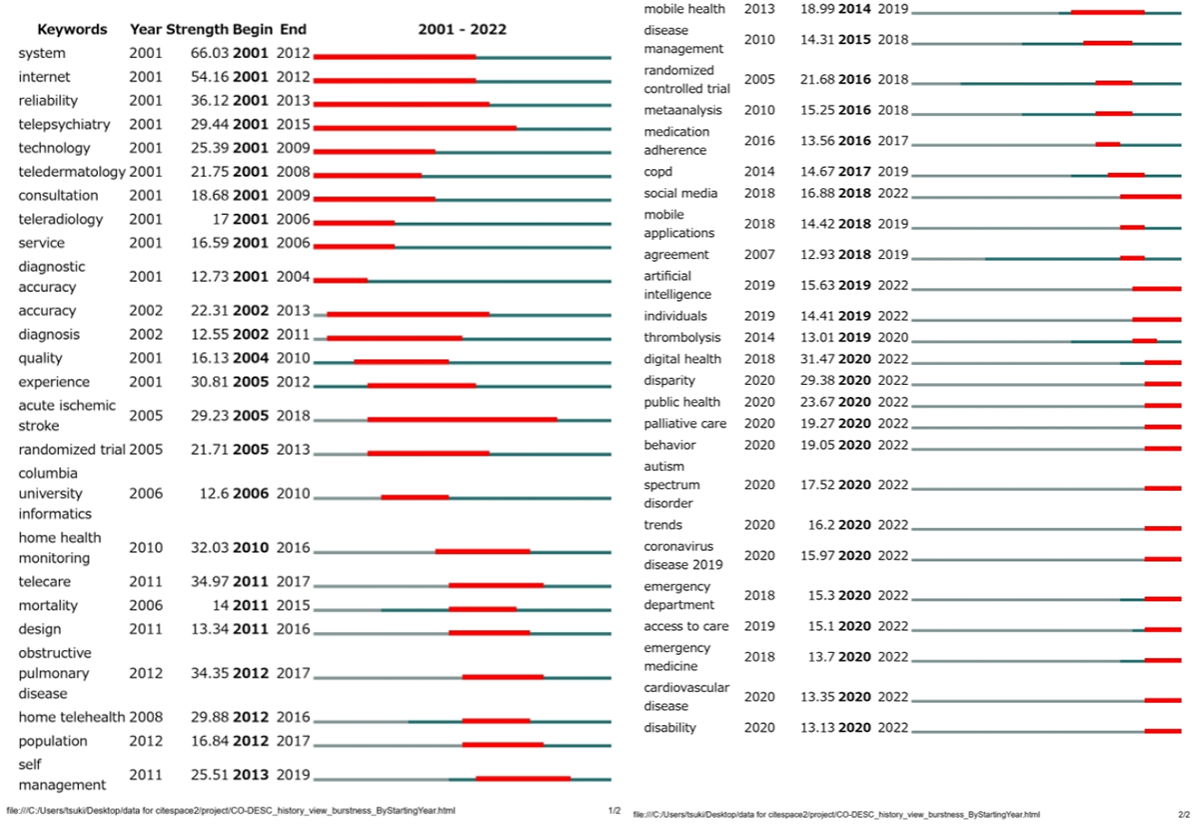
Top 50 international trends with keywords from the co-occurrence network in telemedicine.

Figure 7 shows the bursts of the top 50 global telemedicine keywords between 2001 and 2022. International trends in telemedicine have generally undergone 3 stages of transformation: early focus, mid-term evolution, near-term focus.

Keywords such as “system,” “telepsychiatry,” and “technology” experienced citation explosions between 2001 and 2012, which reflect the early days of telemedicine, system building, and technology applications in specific fields (eg, telemental health). From 2013 to 2017, there was a rapid increase in citations with keywords such as “telecare,” “self-management,” and “mobile health,” which indicates that telemedicine was beginning to become more personalized as mobile technology became popular and self-management tools developed. This was an indication toward development in the direction of globalization and mobility. Beginning from 2018, there was an explosion of citations for keywords such as “social media,” “disparity,” and “emergency medicine,” possibly due to the increased role of social media in health communication, which heightened concerns of inequalities in medical services and increased demand for remote emergency medical services. Beginning from 2020, pandemic-related keywords, such as “COVID-19,” “coronavirus disease,” and “emergency department,” showed significant citation explosions, which highlights the profound impact of the new coronavirus pandemic on telemedicine research and practice. Telemedicine’s focus underwent drastic changes from the initial technical foundation and system construction to the integration of mobile and self-management tools and application of social media during the pandemic.

## Discussion

### Principal Findings

This study revealed significant insights into the evolution and status of telemedicine research globally, with a special focus on China. The primary findings indicate a substantial increase in telemedicine-related publications over the years, particularly noting a surge from 2020 to 2022, which aligns with the COVID-19 pandemic. This increase reflects the growing global and regional recognition of telemedicine as a critical component of health care systems, especially during times of crisis. We identified a total of 25,333 telemedicine-related research papers published between 2001 and 2022, with the United States, the United Kingdom, and Australia leading in publication numbers. However, China's contribution and global ranking in telemedicine research significantly improved after 2020, showcasing the country's rapidly growing interest and efforts in this field.

### COVID-19 and Telemedicine Research

Overall, the development of communication and professional medical technologies made it easier for many people to access medical services [42]. Both international and Chinese trends steadily increased from 2001, according to literature on telemedicine. However, after 2010, the rapid development of smartphones and mobile technologies enabled the further development of telemedicine research [43]. The COVID-19 pandemic significantly influenced and increased the amount of telemedicine research and number of publications from 2020 to 2022. The COVID-19 pandemic resulted in a significant increase in telemedicine use across medical specialties and geographic locations [44]. Telehealth services expanded rapidly, with a significant shift toward virtual care. This shift was well received by patients and health care providers and had high patient satisfaction scores [45]. Additionally, the pandemic accelerated the adoption of telemedicine in clinical practice across various medical disciplines, such as dentistry, pediatric care, endocrinology, rheumatology, and sports medicine [46]. The widespread adoption of telemedicine facilitated continuity of care for patients and highlighted the potential for telemedicine to be integrated into health care services long after the pandemic [47]. In addition, telemedicine led to positive outcomes, such as reduced hospitalizations and emergency department visits, which demonstrated its effectiveness in remotely managing various medical conditions [48].

### Potential Possibilities in Telemedicine Research

China’s top researchers in telemedicine were actively engaged in research in various medical fields. Specifically, research conducted in western China demonstrated the feasibility, acceptability, and effectiveness of telemedicine during the COVID-19 pandemic. This highlighted the significant contribution of researchers in this region toward the advancement of telemedicine [49]. In addition, pilot studies focused on the evaluation of the utilization rate and cost-effectiveness of telemedicine programs in western China, which indicated that researchers were actively involved in the implementation and evaluation of telemedicine programs [50]. Simultaneously, research on the application of telemedicine in Gansu Province, China, emphasized that developed cities, such as Beijing, Shanghai, and Guangzhou, had telemedicine consultation centers and successfully managed diseases, such as hypertension and diabetes [51]. Xu et al [52] examined regional heterogeneity in the application and effect of telemedicine in rural primary care centers in China and revealed the diverse implementation and impacts of telemedicine in different regions.

Overall, research by Chinese scholars in telemedicine spanned across many regions, which included western China, Gansu Province, and rural areas. This demonstrated comprehensive and extensive participation in advancing telemedicine practices and technologies.

From a Chinese perspective, Figure 3 shows a significant increase in the number of Chinese publications in telemedicine research over time, especially after 2020, which was consistent with global trends. This growth trend demonstrates China's increasing interest and investment in telemedicine research, which reflects active research activities and potential research momentum. Simultaneously, Table 1 shows China’s global ranking in telemedicine research publications. China’s global ranking is rising, which shows that its research is valued both domestically and internationally. Moreover, among the multiplier effects of publications in the top 10 countries in Table 1, China’s multiplier effect was greater than that of the other countries, which further emphasizes its research potential.

### International Coauthorship in Telemedicine

From 2001 to 2022, the United States was a central node in telemedicine research and had extensive cooperative relationships with other countries. During this period, China gradually developed from a lower degree centrality and lower betweenness centrality to a lower degree centrality and higher betweenness centrality. This indicates that its role as an information dissemination medium in the international collaboration network was small; however, the number of cooperating countries increased. International influence was improved to a certain extent. South American countries (mainly Brazil), western European countries (mainly Italy), and African countries (mainly Egypt and countries in west Africa) all grew in influence. China is currently conducting relatively close cooperative research with Asian and African countries, led by Egypt and countries in west Africa. However, joint research with South American countries (mainly Brazil) and western European countries (mainly Italy) remains relatively slow. These countries are potential research collaborators. Simultaneously, the United States, Australia, the United Kingdom, and Canada were the most influential countries and maintained close cooperation.

### Research Hot Spots in Telemedicine

Between 2001 and 2019, “care” and “health” became the largest research nodes in telemedicine literature, which reflected that health care was the core of telemedicine research. Technology played a significant role in telemedicine, particularly in mHealth and eHealth applications. Telemedicine research in China focused on the management and application of technology. User acceptance, mobile technology, health information, and data analytics were key points in Chinese studies, which reflects China's special emphasis on telemedicine policy and data management. From 2020 to 2022, “COVID-19” and “pandemic” become prominent keywords in global telemedicine research, which indicates that the pandemic greatly affected telemedicine research and practice. This forced researchers to focus on various areas, such as pandemic management, public health, medical disparities, and digital health. Mental health, urgent care, and data analytics were also areas of focus, which indicates the impact of the pandemic on the demand for telehealth services and technology. Simultaneously, the emergence of keywords shown in Figure 7 reflects the forefront of the research field. The emergence of some keywords, such as “big data” and “artificial intelligence,” indicates that telemedicine research was beginning to integrate advanced technologies, which could potentially completely change the field.

Simultaneously, the world is working on telemedicine for all ages. However, as far as Chinese keywords were concerned, relatively few studies focused on telerehabilitation for children and older adults. In addition, few studies examined telemental health for children. China should also consider strengthening telemedicine research across all age groups. The world's latest telemedicine research direction is toward the development of artificial intelligence, digital medicine, and individualization. China has also conducted extensive research in these fields. In the future, we will continue to deepen research on artificial intelligence and digital medicine in telemedicine.

### Future Prospects for Research in Telemedicine

This study demonstrated China’s significant development in the research field of telemedicine. However, betweenness centrality remained low, which indicates the need for Chinese researchers to seek opportunities that allow them to be the mediators while bonding in smaller subnetworks.

Through this analysis, we found that telemedicine research is likely to focus on improving service efficiency and quality with the advancement of technology, especially in artificial intelligence and mHealth. Future telemedicine research should explore the application and integration of these technologies in the medical field. Personalized medicine and increased patient engagement are expected to be crucial. As international collaboration increases, future telemedicine research should place greater emphasis on knowledge sharing and collaboration internationally. Special keywords, such as “lockdown” and “tracing,” were revealed in the co-occurrence analysis for China, which suggests that telemedicine research also targeted specific regional, social, and cultural factors. This demonstrates that solutions must be identified for each culture, region, and country when it comes to mitigating social problems and health challenges through telemedicine development. Policy and regulation will remain an important topic in telemedicine research, particularly regarding data security and privacy protection.

Simultaneously, the impact of the COVID-19 pandemic on telemedicine will be reflected in future research, including the exploration of public health emergency management, vaccination, pandemic monitoring, and remote patient management. Future research is expected to explore how telemedicine technology can help respond to global health crises. Different cultural and social contexts may bring different acceptance and needs for telemedicine. Considering how to provide medical services that are more adaptable to different cultural and social environments will be a direction for future research.

### Limitations

This study has 4 limitations. First, we only collected bibliometric data from the Web of Science database and excluded other search engines, such as Scopus, PubMed, IEEE, and Google Scholar. Second, we extracted only articles published in English. Third, although we aimed to identify research trends in telemedicine in China, we obtained limited articles on telemedicine, as we only used those published in Web of Science. Fourth, some data in the literature data collected through Web of Science could be changed due to electronic publishing or document editing and publication time. Hence, this may impact the data analysis results.

### Conclusions

This study provides the latest trends in telemedicine research, demonstrates that telemedicine research has considerable potential in China, and provides directions for future development. Simultaneously, China’s future remote medical research collaboratives and research areas are also of reference value. Although this study shows significant growth in telemedicine research in China, it points out that China's status as a center for international collaboration is still low. In the future, telemedicine research is likely to focus on improving service efficiency and quality, particularly by leveraging technological advances in artificial intelligence and mHealth. In addition, personalized medicine and increased patient engagement will be important trends in the future.

## Appendix

Multimedia Appendix 1 List of degree and betweenness centrality by country from 2001 to 2019.

Multimedia Appendix 2 List of degree and betweenness centrality by country from 2020 to 2022.

## Abbreviations

mHealth: mobile health
